# Dataset of physico-chemical water parameters, phytoplankton, flora and fauna in mangrove ecosystem at Sungai Kertih, Terengganu, Malaysia

**DOI:** 10.1016/j.dib.2022.108096

**Published:** 2022-03-26

**Authors:** Siti Mariam Muhammad Nor, Maisarah Jaafar, Nik Mohd Shibli Nik Jaafar, Nurul Shahida Redzuan, Wan Bayani Wan Omar, Muhammad Yazid Deraman, Siti Nur Syasya Nadhirah Azli, Nur Adilla Shehrom, 'Ainna Mahyudin, Nor Ain Bahari, Nurul Nadhirah Zulkafli, Nur Faezati Jaafar, Siti Nur Syaza Ma'ad, Mohammad Izuan Mohd Zamri, Azrun Amirudin, Nur Ain Abdullah, Nurulhuda Zakaria

**Affiliations:** a Faculty of Science and Marine Environment, Universiti Malaysia Terengganu, 21030 Kuala Nerus, Terengganu, Malaysia; b Malaysian Nature Society, ecoCare Environmental Education Centre, Kampung Labohan, 24300 Kertih, Kemaman, Terengganu, Malaysia; c Coastal and Mangrove Mitigation Group (CoasMag), Faculty of Science and Marine Environment, Universiti Malaysia Terengganu, 21030 Kuala Nerus, Terengganu

**Keywords:** Bivalve, Brackish, Coastal, Crustacea, Herpetofauna, Phytoplankton, Water parameter, Sediment

## Abstract

This data article presents the composition of selected physico-chemical water parameters, phytoplankton, flora and fauna in Sungai Kertih, Terengganu, Malaysia. Sungai Kertih is surrounded by mangrove forest and located nearby to a coastal hill namely Bukit Labohan that supports vast biological resources to its adjacent inhabitants. Therefore, a biodiversity and environmental assessment was conducted in Sungai Kertih from 19–21 August 2019 to document the biodiversity and physico-chemical water parameters of the river. The dataset show that Sungai Kertih is occupied by 14 tree mangrove species, 43 phytoplankton species, 21 bivalve species, 10 crustacean species, five amphibian species and eight reptilian species. The obtained physico-chemicals water parameters data were the value of pH, total dissolved solids, dissolved oxygen, temperature, salinity and electrical conductivity. Understanding the influence of physical and chemical properties on biological resources in coastal ecosystem is one of important in river conservation and management practices. Thus, this baseline dataset can be utilized for further reference and monitoring the health of the environment in the mangrove ecosystem.

## Specifications Table

**Table:** 

Subject	Environmental Science
Specific subject area	Ecology, and Hydrology and Water quality
Type of data	Table Figure
How data were acquired	Water quality parameters: pH, total dissolved solids (TDS), dissolved oxygen (DO), temperature, salinity, and electrical conductivity were measured in-situ using a YSI multi-parameter probe. Plant: The mangrove tree data were obtained through mangrove inventory study by setting up a square plot 200 m × 10 m (0.2 ha). All tree species in the plot were labelled, enumerated and identified. Phytoplankton: Water samples were collected using a 30 mm wiremesh plankton net. Phytoplankton cells were extracted from the water by means of centrifugation at 2300 rpm for 15 minutes. For enumeration and identification, diatoms cells were sedimented in a sedimentation chamber after being washed following the acid washed protocol [1]. Diatoms cells were enumerated up until 200 cells and represented as relative abundance data. The cells were identified to the lowest possible taxa, to genus or species level. Mollusc (bivalves & gastropods) and Crustacean: Animals were hand collected during low tide within sampling subplots. All individual collected were counted, sorted and identified in lab. Herpetofauna (amphibians & reptiles): The animals were captured with a sweep net, a snake tong or by hand grabbing. The body parts of the animal were measured including snout-vent length (SVL), head length (HL), head width (HW), tibia length (TBL), thigh length (THL), finger length (FL), tail length (TL) using a Vernier caliper and the animal was weighted (W) using a compact balance.
Data format	Raw
Description of data collection	Descriptive abundance by pool per taxa. The species recorded list then was compared to the International Union for Conservation of Nature Red List (IUCN Red List) database to determine the exact conservation status of species in Sungai Kertih.
Data source location	Institution: Malaysian Nature Society City/Town/Region: Kertih, Kemaman, Terengganu Country: Malaysia Latitude and longitude (and GPS coordinates, if possible) for collected samples/data: 4.5263 °N, 103.4441 °E.
Data accessibility	Repository name: Harvard Dataverse, V2 Data identification number: doi:https://doi.org/10.7910/DVN/GHOIM4 Direct URL to data: https://doi.org/10.7910/DVN/GHOIM4

## Value of the Data

• The data is constructed from the selected phytoplankton, flora and faunal abundance together with the physico-chemical water parameters. Thus, the data highlight local species record and contributes to establish the first species checklist of Sungai Kertih, Terengganu. Both the water parameters and the organisms are crucial as a dataset that can well represent the status of the investigated ecosystems health.
• The physico-chemical water parameters are very important to get an exact idea about the quality of water and can be used to compare different physico-chemical parameter values with standard values. The physico-chemical water parameters data is important to further investigate the effects on organism productivity and to understand the factors that play a role in the distribution pattern of organisms.
• The presented organism's data in this study are all of the group that widely be used as bioindicators of the aquatic and mangrove ecosystems, which is believed can be a valuable data for present and future reference. These bioindicators can be used to assess the quality of the environment and are also an important tool for detecting changes in the environment, and their subsequent effects on human society.
• The taxa checklist could be used by scientific community to evaluate and compare with the taxa present in other mangrove areas or other ecosystems, evaluating Sungai Kertih biodiversity and environment health, and identify the important species to protected and conserved. The data can be useful for further researches that deal with any coastal scenarios or coastal management research.
• The availability of data allow further insight into which physical or chemical factors influence the onset duration and visibility of selected taxa in mangrove ecosystem, contrasting terrestrial environments, as well as at areas with substantial human encroachment. The knowledge of physico-chemical water parameter changes in mangrove areas is an important component for monitoring and management activities concerned with land use to detect the natural seasonal changes in the brackish areas and to identify the changes from anthropogenic input. The organisms of different taxa may be used as indicators of biological monitoring in the brackish and coastal area.
• The data strengthen the need to preserve mangrove forests and to restore those degraded to guarantee the provision of goods and services needed to support the biodiversity and functioning of wide portions of tropical areas.

## 1 Data Description

**Fig. 1:**
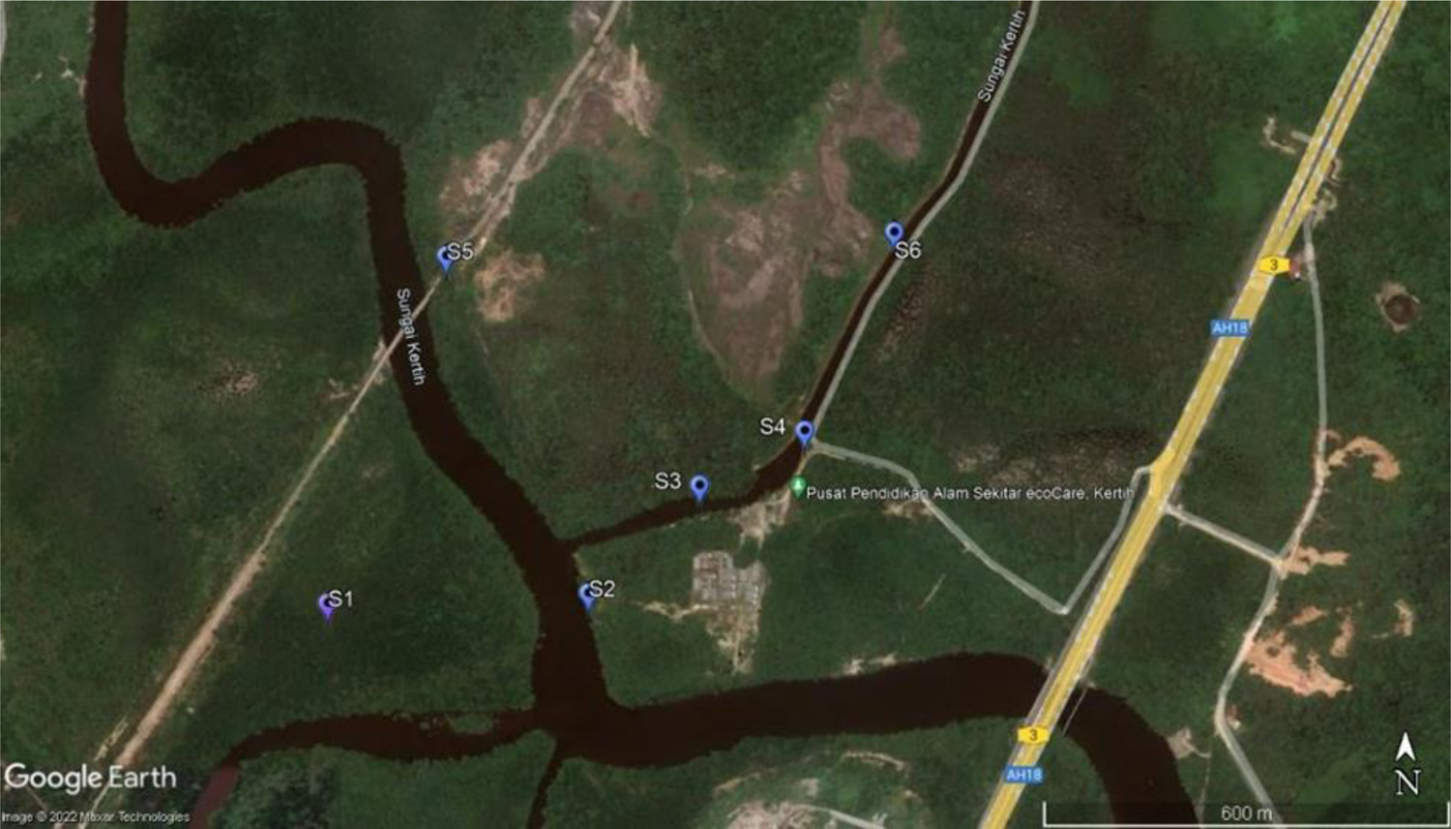
Map showing the sampling locations (Station 1; S1 - Station 6; S6) in Sungai Kertih mangrove forest in Peninsular Malaysia (source: Google Map).

The mangroves of Sungai Kertih are located in Kemaman District, Terengganu, Peninsular Malaysia (Fig. 1). A morphological feature of the area is the Sungai Kertih, a coastal river approximately 23 km long that originates in hilly terrain at about 350 m above sea level, before running through the mangroves and entering the sea just north of Kertih Town [3]. The data presented in this article were primary data on selected physico-chemical water parameters together with the flora data (mangrove tree species), protist (phytoplankton) and faunal taxa data namely bivalve, crustaceans, amphibians and reptiles in Sungai Kertih mangrove forest.

Table 1 demonstrated the water quality parameters of water samples from Sungai Kertih. The range for water quality parameters in all sites (Station 2 - Station 4) is as follows: temperature, 28.62 - 30.37°C; pH, 5.47 - 6.05; dissolved oxygen, 2.35 - 4.09 g/l; conductivity, 38.77 – 48.69 mS/cm; total dissolved solids, 22.94 - 27.56 g/l and salinity, 22.14 - 27.33 ppt. All raw data linked to Table 1 were shared via a data repository [2].

**Table 1:** Physico-chemical water parameters of Sungai Kertih.

Location	Station 2	Station 3	Station 4
Parameter	Mean ± SD	Mean ± SD	Mean ± SD
Temperature (°C)	28.89 ± 0.61	28.62 ± 0.13	30.37 ± 1.04
Conductivity (mS/cm)	48.69 ± 5.67	42.42 ± 0.91	38.77 ± 8.51
Total Dissolved Solid (g/l)	27.56 ± 0.91	25.80 ± 0.53	22.94 ± 8.51
Salinity (ppt)	27.33 ± 0.96	25.21 ± 0.59	22.14 ± 5.89
Dissolved Oxygen (mg/l)	2.35 ± 0.19	3.88 ± 0.37	4.09 ± 0.78
pH	5.90 ± 0.09	5.47 ± 0.18	6.05 ± 0.05

The checklist in Table 2 showed the species composition and diversity of mangrove tree from natural mangrove forest in Sungai Kertih. A total of 14 mangrove species belonging to eight families were identified with Rhizophoraceae being the largest plant family as represented by six species. 86% of species listed are exclusive mangrove species or true mangroves.

**Table 2:** Mangrove species composition along Sungai Kertih including their exclusivity classification and the IUCN Red List conservation status with the year of papers were published.

Family	Species	Exclusivity	Local Name	Conservation Status	Year Published
Acanthaceae	*Avicennia alba* Blume	Exclusive	Api-api putih	Least Concern	2010

Arecaceae	*Nypa fruticans* Wurmb.	Exclusive	Nipah	Least Concern	2010

Euphorbiaceae	*Excoecaria agallocha* L.	Exclusive	Buta-buta	Least Concern	2010

Lythraceae	*Sonneratia caseolaris* (L.) Engler.	Exclusive	Berembang	Least Concern	2010

Malvaceae	*Heritiera littoralis* Aiton	Exclusive	Dungun	Least Concern	2010
	*Talipariti tiliaceum* (L.) Fryxell.	Non-exclusive	Baru-baru	Least Concern	2019

Meliaceae	*Xylocarpus granatum* (L.) Koenig	Exclusive	Nyireh bunga	Least Concern	2010

Rhizophoraceae	*Rhizophora apiculata* Blume	Exclusive	Bakau minyak	Least Concern	2010
	*Rhizophora mucronata* Lam.	Exclusive	Bakau kurap	Least Concern	2010
	*Bruguiera gymnorhiza* (L.) Lam. *Ex* Savigny	Exclusive	Tumu merah	Least Concern	2010
	*Bruguiera cylindrica* (L.) Blume	Exclusive	Berus-berus	Least Concern	2010
	*Bruguiera parviflora* (Roxb.) Wight & Arn.ex	Exclusive	Lenggadai	Least Concern	2010
	*Kandelia candel* (L.) Druce.	Exclusive	Bakau lilin	Least Concern	2010

Sapotaceae	*Planchonella obovata* (R.Br.) Pierre	Non-exclusive	Menasi	Not Evaluated	-

A total of 56 phytoplankton species were recorded at Station 2, Station 3 and Station 4 in Sungai Kertih (Table 3). Of the total species, 46 species belong to the division of Bacillariophyta; four species belong to four genera of Chlorophyta; five species from three genera of division Dinophyta; and only one Cyanophyceae species was recorded, which was the *Oscillatoria tenuis*. The chain-forming *Chaetoceros curvisetus* of the order Chaetocerotales was the most abundant species and recorded in all of the three stations, but with the highest occurrence at Station 2. Whereas, at Station 2, *Frustulia vulgaris* was reported with the highest abundance of 24.92 ± 3.44%. All raw data linked to Table 3 were shared via a data repository [2].

**Table 3:** Relative abundance (RA) (%) of phytoplankton at three different stations. The RA values for each station were the averaged of two sampling days data (triplicates samples in each sampling day).

		Stations		
No.	Taxa	Station 2	Station 3	Station 4
	Division Bacillariophyta			
1	*Actinoptychus undulatus*	0.25 ± 0.29		1.02 ± 0.42
2	*Amphora* sp.			0.42 ± 0.17
3	*Asterolampra* sp.			0.21 ± 0.09
4	*Bacteriastrum delicatulum*	0.08 ± 0.02		
5	*Bacteriastrum furcatum*	0.08 ± 0.02		
6	*Bacteriastrum* sp.1	6.67 ± 2.70	5.75 ± 3.59	2.72 ± 1.11
7	*Bacteriastrum* sp.2		0.17 ± 0.26	
8	*Chaetoceros constrictus*		0.08 ± 0.01	5.81 ± 2.37
9	*Chaetoceros curvisetus*	27.08 ± 5.07	7.50 ± 5.16	8.80 ± 3.59
10	*Chaetoceros* sp.	5.33 ± 3.75		2.42 ± 0.99
11	*Climacodium frauenfeldianum*	2.50 ± 0.63	0.92 ± 1.11	1.28 ± 0.52
12	*Coscinodiscus radiatus*			0.58 ± 0.24
13	*Coscinodiscus* sp.1	1.33 ± 0.17	1.58 ± 1.11	1.21 ± 0.49
14	*Coscinodiscus* sp.2	0.17 ± 0.10	0.25 ± 0.61	0.82 ± 0.33
15	*Cocconeis placentula*	0.08 ± 0.10		
16	*Cyclotella meneghiniana*	0.08 ± 0.10	0.08 ± 0.20	0.20 ± 0.08
17	*Cymbella* sp.	0.50 ± 0.20	0.33 ± 0.61	0.61 ± 0.25
18	*Diploneis* sp.	0.25 ± 0.17		0.41 ± 0.17
19	*Ditylum brightwellii*	0.08 ± 0.10		0.21 ± 0.09
20	*Eucampia zodiacus*		0.33 ± 0.82	
21	*Eunotia valida*	0.92 ± 0.48		
22	*Frustulia vulgaris*	15.17 ± 0.00	24.92 ± 3.44	3.13 ± 1.28
23	*Fragilaria* sp.		1.25 ± 1.75	3.90 ± 1.59
24	*Guinardia delicatula*	7.17 ± 3.15	1.92 ± 3.44	5.57 ± 2.27
25	*Guinardia flaccida*	4.50 ± 0.93	2.92 ± 2.69	4.83 ± 1.97
26	*Guinardia striata*	1.08 ± 1.27	3.67 ± 6.91	4.89 ± 1.99
27	*Gyrosigma scalproides*		0.17 ± 0.41	0.20 ± 0.08
28	*Gyrosigma* sp.			0.87 ± 0.27
29	*Navicula radiosa*		0.25 ± 0.03	
30	*Navicula peticolasii*		0.08 ± 0.03	
31	*Nitzshia longissima*		0.08 ± 0.02	0.20 ± 0.08
32	*Nitzschia epithemoides*	0.75 ± 0.21	0.17 ± 0.41	
33	*Odontella sinensis*			0.42 ± 0.17
34	*Pinnularia acuminata*	0.50 ± 0.10	1.00 ± 0.88	
35	*Pinnularia* sp.	1.08 ± 0.88		
36	*Pleurosigma directum*	0.08 ± 0.10	0.08 ± 0.03	
37	*Pleurosigma elongatum*		0.33 ± 0.16	
38	*Pleurosigma* sp.			1.21 ± 0.49
39	*Rhizosolenia alata*			1.02 ± 0.42
40	*Rhizosolenia imrbicata*	0.83 ± 0.48		1.87 ± 0.27
41	*Rhizosolenia striata*	5.08 ± 0.40	1.08 ± 0.08	0.66 ± 0.27
42	*Skeletonema* sp.	0.33 ± 0.10		0.66 ± 0.27
43	*Strauroneis producta*			0.49 ± 0.20
44	*Synedra ulna*		6.75 ± 1.72	2.09 ± 0.85
45	*Triceratium favus*	0.58 ± 0.58	0.08 ± 0.01	0.42 ± 0.17
46	*Zygoceros atlanticus*	0.75 ± 0.15		

	Division Chlorophyta			
47	*Closterium* sp.			0.20 ± 0.08
48	*Mougetia* sp.	7.50 ± 1.34	10.00 ± 2.61	5.21 ± 2.13
48	*Pleurococcus miniatus*	7.83 ± 3.11	17.00 ± 3.12	4.77 ± 1.95
50	*Ulothrix* sp.	0.50 ± 0.10	5.42 ± 1.30	4.46 ± 1.82

	Division Dinophyta			
51	*Ceratium* sp.	0.08 ± 0.02	0.33 ± 0.10	0.41 ± 0.17
52	*Dinophysis* sp.	0.08 ± 0.10		0.41 ± 0.17
53	*Protoperidinium pallidum*	0.42 ± 0.11	0.08 ± 0.01	0.80 ± 0.33
54	*Protoperidium cinctum*	0.08 ± 0.02		0.42 ± 0.17
55	*Protoperidium* sp.	0.17 ± 0.20	0.25 ± 0.02	0.52 ± 0.21

	Division Cyanophyta			
56	*Oscillatoria tenuis*		4.83 ± 0.51	2.80 ± 1.14

Table 4 demonstrated the distribution and abundance of mollusk and crustacea in Sungai Kertih mangrove area. Four species of bivalve from four families (Class Bivalvia), seven species of gastropod from four families (Class Gastropoda) and 10 species of crustacea from three families (Class Malacostraca) were recorded from six stations (Station 1 - Station 6). The highest abundance was recorded in Station 5 (S5) with 330 individuals while the highest abundance from all six stations was recorded by *Enigmonia* sp. with 177 individuals.

**Table 4:** Species of mollusks and crustaceans with distribution and its abundance collected and identified at six stations along Sungai Kertih.

Class	Family	Species	Station 1	Station 2	Station 3	Station 4	Station 5	Station 6	Total
Bivalvia	Cyrenidae	*Geloina expansa*	0	1	0	9	8	10	28
	Veneridae	*Marcia japonica*	0	0	8	0	0	0	8
	Veneridae	*Meretrix meretrix*	0	0	21	0	0	0	21
	Anomiidae	*Enigmonia* sp*.*	0	0	0	41	130	6	177

Gastropoda	Potamididae	*Telescopium telescopium*	6	38	0	15	21	2	82
		*Cerithidea* sp.	10	21	6	20	87	29	173
	Neritidae	*Nerita balteata*	6	0	0	6	1	0	13
	Ellobiidae	*Cassidula rugata*	15	0	0	8	32	1	56
		*Cassidula nucleus*	7	12	0	4	19	0	42
	Littorinidae	*Littoraria carinifera*	32	3	16	8	13	3	75
		*Littoraria* sp.	1	9	0	5	2	1	18

Malacostraca	Sesarmidae	*Sersarmidae* sp.	4	6	0	5	6	1	22
		*Sarmatium germaini*	3	3	0	1	4	0	11
		*Perisesarma eumolpe*	4	1	0	0	0	0	5
		*Perisesarma* sp.	1	0	0	0	0	0	1
		*Episesarma mederi*	0	0	0	0	1	0	1
		*Haberma* sp.	2	5	0	0	2	0	9
		*Labuanium politum*	1	0	0	0	3	0	4
	Ocypodidae	*Uca forcipata*	0	1	0	0	0	0	1
	Varunidae	*Metaplax elegans*	0	4	0	0	1	0	5
		*Metaplax* sp.	0	3	0	0	0	0	3
		Total	92	107	51	122	330	53	755

A total of 18 individuals from five species and three families of amphibians were documented at Sungai Kertih (Table 5). *Fejervarya cancrivora* (crab-eating frog) was the most dominant species with a total of 11 individuals, followed by *F. limnocharis* (two individuals), *Phrynoidis asper* (two individuals), *Duttaphrynus melanostictus* (two individuals) and *Polypedates leucomystax* (one individual).

**Table 5:** The morphological data of the amphibians including the snout-vent length (SVL), head length (HL), head width (HW), thigh length (THL), tibia length (TBL), finger length (FL), mass, and the IUCN Red List conservation status with the year of papers were published.

Family	Species	SVL (cm)	HL (cm)	HW (cm)	THL (cm)	TBL (cm)	FL (cm)	Mass (g)	Conservation Status	Year Published
Bufonidae	*Phrynoidis asper*	6.5	2.0	2.5	2.4	2.3	3.3	22	Least Concern	2021
		6.2	1.9	2.2	2.4	2.1	3.3	21		
	*Duttaphrynus melanostictus*	7.5	2.7	2.4	2.6	2.3	3.7	52	Least Concern	2004
		6.8	1.9	2.2	2.5	2.1	3.5	36		

Dicroglossidae	*Fejervarya cancrivora*	3.7	1.3	1.2	1.8	2.0	2.7	6	Least Concern	2004
		5.2	1.7	1.6	2.1	1.8	3.6	12		
		3.4	1.2	1.0	1.4	1.3	2.5	3		
		5.2	1.4	1.5	2.2	2.0	3.8	13		
		3.9	1.3	1.3	1.7	1.7	2.9	6		
		5.2	1.3	1.4	2.2	2.0	3.6	13		
		5.8	1.8	1.6	2.0	2.3	3.8	17		
		5.8	2.0	1.7	2.3	2.7	4.0	18		
		7.1	2.2	1.9	2.6	3.0	4.5	38		
		7.5	2.5	2.4	2.8	3.1	5.1	53		
		7.4	2.3	2.1	2.6	2.9	4.8	44		
	*Fejervarya limnocharis*	4.1	1.5	1.3	1.4	2.0	3.0	17	Least Concern	2004
		3.8	1.3	0.9	1.7	1.7	2.9	12		

Rhacophoridae	*Polypedates leucomystax*	4.3	1.4	1.2	2.2	2.1	2.7	27	Least Concern	2004

Five families of reptiles namely Agamidae, Gekkonidae, Homalopsidae, Scincidae and Varanidae were found at Sungai Kertih mangrove forest (Table 6). A total of 19 individuals from eight species of reptiles were identified at Sungai Kertih. Snake species from family Homalopsidae recorded 47% from the total number of individuals found, followed by Agamidae (32%), Scincidae (11%), Gekkonidae (5%) and Varanidae (5%).

**Table 6:** The morphological data of the reptiles according to the species including the tail length (TL), snout-vent length (SVL), mass, and the IUCN Red List conservation status with the year of papers were published.

Family	Species	TL (cm)	SVL (cm)	Mass (g)	Conservation Status	Year Published
Agamidae	*Leiolepis belliana*	23.0	13.0	83	Least Concern	2019
		23.5	12.5	61		
		27.2	13.7	74		
		29.6	15.0	106		
	*Calotes versicolor*	18.2	12.5	60	Least Concern	2021
		25.5	13.5	72		

Gekkonidae	*Hemidactylus frenatus*	5.5	4.5	23	Least Concern	2021

Homalopsidae	*Hypsiscopus plumbea*	3.1	14.8	118	Least Concern	2010
		3.1	13.2	93		
		2.6	14.9	113		
		2.2	11.5	92		
		2.4	13.3	98		
		5.8	30.9	300		
		3.2	17.7	123		
	*Cerberus rynchops*	12.5	46.8	79	Least Concern	2010
		13.7	44.6	73		

Scincidae	*Eutropis multifasciata*	-	-	-	Least Concern	2018
	*Eutropis rudis*	5.6	4.3	13	Least Concern	2021

Varanidae	*Varanus salvator*	-	-	-	Least Concern	2021

## 2 Experimental Design, Materials and Methods

During fieldwork, water quality parameters were recorded between 10 am and 11 am. The water samples were obtained from soil porewater at 30 cm depth. In each location, the physico chemical parameters of surface water namely, pH, total dissolved solids (TDS), dissolved oxygen (DO), temperature, salinity, and electrical conductivity were measured *in-situ* using a YSI multi-parameter probe. The reading for all parameters was taken three times at each sampling site.

A plot with the size of 200 m × 10 m (0.2 ha) was set up randomly in a natural mangrove forest of Sungai Kertih to determine the mangrove species composition. Within the plot, all the tree species were identified *in situ*. However, for the plant species that was difficult to identify *in situ*, the plant specimens i.e. plant leaves and inflorescent were brought back to the laboratory for further identification by an expert assistance and also by referring to the mangrove books [4,5]. The data then were used to list the diversity and composition of mangrove species of the area and also for further species classification based on their exclusivity as referred to [6]. Conservation status of each species also was determined according to the IUCN Red List of Threatened Species.

For phytoplankton, three stations were set up which were the Station 2 (4.525494 °N,103.441899 °E) located in waterway discharge from Kampung Gelugor, Station 3 (4.526935 °N, 103.443382 °E) at replanted mangrove area, and Station 4 (4.527664 °N, 103.444784 °E) at boat parking area. Water samples were collected using a 30 mm wiremesh plankton net. Phytoplankton cells were extracted from the water by means of centrifugation at 2300 rpm for 15 minutes. The cells were acid washed prior to preparation of slides. For enumeration and identification, diatoms cells were sedimented in a sedimentation chamber after being washed following the acid washed protocol [1]. Diatoms cells were enumerated up to 200 cells and represented as relative abundance data. The cells were identified to the lowest possible taxa, to genus or species level.

Samplings of mollusks and crustaceans were conducted during low tide at six stations (Station 1 – Station 6) along Sungai Kertih. In each station, three sampling sub plots size 2 × 2 m were constructed randomly where mollusks samples were collected by hand. For bivalves and crustaceans, a hand rake was used to scoop out burrowing (about 10 cm from surfaces) bivalves and crustaceans in the sediment [7]. The collected mollusks, bivalves and crustaceans were then sorted and identified accordingly [8].

The Visual encounter survey (VES) method was applied to catch amphibians and reptiles. VES is a time-constrained method in which observer sample for species richness and abundance along a survey path [9]. The VES involves sampling of amphibians and reptiles at both daytime and night. These two different survey times have provided an equal temporal representation, so that both diurnal and nocturnal species were observed. At daytime, the VES was conducted between 8.00 am and 11.00 am, and during the night-time between 8.00 pm and 11.00 pm. A wide-beam headlamp was used in searching for amphibians and reptiles at night by walking at the study areas such as along the river and the forest trails [10]. Each of the captured individual's body part was measured using a digital Vernier caliper and weighted using a compact balance. Each of the individuals was identified up to the species level according to [11].

## Ethics Statements

The authors declare that all animal experiments comply with the ARRIVE guidelines and were carried out in accordance with the U.K. Animals (Scientific Procedures) Act, 1986 and associated guidelines, EU Directive 2010/63/EU for animal experiments.

## CRediT Author Statement

**Siti Mariam Muhammad Nor:** Data collection, Supervision (mangrove plant), Original draft preparation*,* Writing – review & editing; **Maisarah Jaafar:** Data collection, Supervision (water parameters), Original draft preparation; **Nik Mohd Shibli Nik Jaafar:** Data collection, Supervision (water parameters), Original draft preparation; **Nurul Shahida Redzuan:** Data collection, Supervision (phytoplankton), Original draft preparation; **Wan Bayani Wan Omar:** Data collection, Supervision (mollusks), Original draft preparation; **Muhammad Yazid Deraman:** Supervision (field work); **Siti Nur Syasya Nadhirah Azli, Nur Adilla Shehrom, 'Ainna Mahyudin, Nor Ain Bahari, Nurul Nadhirah Zulkafli, Nur Faezati Jaafar, Siti Nur Syaza Ma'ad, Mohammad Izuan Mohd Zamri, Azrun Amirudin** and **Nur Ain Abdullah:** Data collection; **Nurulhuda Zakaria:** Data collection, Supervision (herpetofauna), Writing - original draft preparation.

## Declaration of Competing Interest

The authors declare that they have no known competing financial interests or personal relationships which have, or could be perceived to have, influenced the work reported in this article.

## Data Availability

Dataset of physico-chemical water parameters, phytoplankton, flora and fauna in mangrove ecosystem at Sungai Kertih, Terengganu, Malaysia (Original data) (Dataverse).
